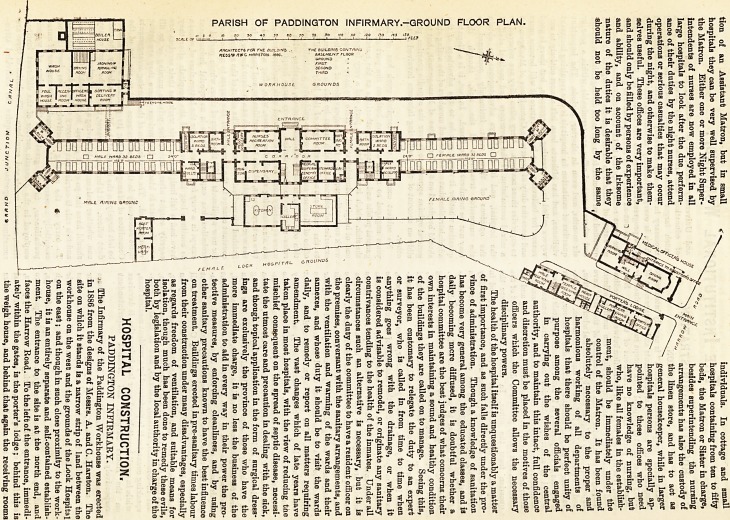# Paddington Infirmary

**Published:** 1892-07-02

**Authors:** 


					HOSPITAL CONSTRUCTION,
PADDINGTON INFIRMARY.
j. The Infirmary of the Paddington Workhouse was erected
in 1886 from the designs of Measrs. A. andC. Harston. The
i site on which ib stands is a narrow strip of land between the
1 workhouse on the west and the grounds of the Lock Hospital
) on the east; and though in such close proximity to the work-
i house, it is an entirely separate and self-contained establish-
1 ment. The entrance to the site is at the north end, and
1 faces the Harrow Road. To the left of the entrance, immedi-
-L? ately within the gates, is the porter's lodge; behind this is
the weigh house, and behind that again the receiving rooms
&* ??
(6 <L
?<
2. 4
?frg
r 5*
5 p*
o o
P 5-
a> g
o- *?
o
p- CQ
?2* ct
P ty
P- ffl
Et ?T=J
?f ?
ct- tn-
^5 DO
H" o"
ct- P"
cr era
(B ?
3 g* 2 4 2. 5*
(B 2 B O 2" ca
B C cf- |-S ? ??I ("d
S- g p* w* o oo cr
? - ? g* B gg ?
<n 2 ^ .. i?i
f-3 ?? g g 2. S"
cr ? g. ?
? " ? 0 5* g b*
p 2 cr w p
? M Q3 P ct- &3
B B B ct- 5-* B* S
ET g p, ? Z ? ?<
? CJ- rt- ? o- a o
g r?- b* d 2 ? M
? S O 5 P ?
? ?? ? S g'OQ B
?- a ? g ?
o g cr g, -? oo
?S s* b ~ o ^
ct-'g B Q. 8? l-h P
B* 2 m ^ ^ p-
a
*1 cs
1-1 ?
2. ?. g
a H3
K 1-1
C ?
g 3
pj .s
T3 P
o o
*. p.
- Q
?3?
S? E? ? B L_,
g-v< f p, H
CD Ct-
g
?
. t-i
^ s
? s
cr f>
I 3
**?* I 2" 2 |
?2fwi?:
p 2" ? O g ? 8
s ^ ? S s
m j"' o ^3 i *i O
g 5* ^ -? ct- H ct-
? ST o g" g- g,
pj i i CD CD M4
X
o
CO
-a
H
>
t-
o
o
2
CO
H
50
c:
o
H
o
2
N
2.SBS&P
CD
o B B
g" ? ?> ?S*
P- g ^ ^
? C & g* gr
P^ ct- r! CD
CD 5jV O 513 00
S> ^ s?
l-l CD
ef tr
g- ?
CD m
CD ii K
1 O 8>
~3:
3 s-i
tr ^
? ?- B
P 2 o
JZ'Z*
?S & ?
? ? B
g p-f
S o
s ? ^
i? ^3 g,
CD
& ** ix.
S?S
CD ?*
E.? &
SI
0*5 <T>
B* 2
ct-
CQ S*
n 2
?S P-
o*
V!
s>
B
P*
f-r- <
*< g^?
S5 M
ct- as g.
rr ?ff.
CD ~ JT* m
? g g. 5
o 2 ??? p
G ? 2 B
S ^ ^
.B ?< 5
8? P<,f
?? 5 ffl
rr- ct- Qj ?
as s i
<
ct-' ? S. ?
1.B g- ??
-.15 ? ?
B Pi Z TO
o ?<5 ? ?
?
tg g g iS
? ? B ?
O ? co ?.
<. p
ft-, >-* Z-
M 0Q O t*~T*
o >. ?-l *<
^ ro p
^ o- B o
ct- CD Pj ^
S3* ? ?
? ? er -?
2 5,^ ?
r c b ? s,
~ CD co M
2 p pr ^ st
C o ? o ? ?
bj CD CfQ ? CD CD
ZD
oa p- -
CD Q O
5 f3 *
|! 2 S &
2 -gr-
S,? &?
""O St)
5" s?* ?
JL, o 3.
g"00 -1
g ?
_ OS
S, C3-
i-n 0
ct- P
?s
f'
4 B-tJ S3- TO
i8> O O ?
K <1 S* ? B
o a B ?g ?
i?? M ? S- p
B Q. s> t"
sr o ??1
? - " B-
CJ a ? ^ ?
o B
2 oq -
B p ?<
? to
2 ? o o
{Hi p4 H) H
g g a
B B Cr1
P4 Pi <d
i? B S
b'to ?
B S 2
a B
ta t3 T3
2 ? ? ST ?
~ ? 2 2.
? g* B
? P ct-
oo ?
S 85 g
B j?. Pi
g' B*
? O K
P. M K
J1 *1
ct- S
g" 00* J3
s?
pj CQ
O B
S
P R
ct- ?t" w
S 1- B
B g.
0 B g
1 TO S-
M f? S3"
5' o m
B ?
^ ? s
B B B
I* ? a.
rl KB
TO s>
? -< h
PARISH OF PADDINGTON INFIRMARY.?GROUND FLOOR FLAN.
July 2, 1892. THE HOSPITAL. 223
for patients. Under the receiving room for female patients is a
disinfecting apparatus. To the right of the entrance is the
residence of the Medical Superintendent. The main
building consists of a central administrative block, two
Ward pavilions, north and south, and a projecting wing
to the east, which is one storey in height only. The
central part has a basement and four upper storeys, a
portion only being carried up an additional floor. The ward
wings are four storeys in height with a basement in addition.
The central block contains in the basement, Btore rooms ; mess
rooms for nurses, scrubbers, and servants ; larders and
cellars. The one-storey wing referred to is on the level of this
floor, and contains the kitchen and scullery and a large store
room; all these are top-lighted. On the ground floor of the
central block is the entrance for officers, with nurses' recrea-
tion room and committee-room to right and left. At the back
are dispensary and drug store, Medical Superintendents' office,
stewards' office, and lavatory. On the first floor are the
Matron's office,her bedroom,sitting-room,and bath-room; the
assistant Medical Officer's sitting-room, bedroom, and bath-
and bed-room, the clinical assistant's sitting-room, and the
Matron's linen store. On the second floor are nine Bingle and
two double bed-rooms for nurses, sitting-room and bed-room
for assistant Matron, bed-room for cook, and bath-room,
lavatory, and water-closet for- nurses and servants. The
third floor contains five single and five double bed-rooms for
nurses, one single and two double bed-rooms for servants.
On the fourth floor are two bed-rooms for nurses. In all these
bed-rooms, and also in the bath-rooms, fireplaces are pro-
vided. The patients' entrances to the wards are on the west
side, and between the central block and the wardjpavilions ;
that for female patients being to the north, that for male
patients to the south. In each case immediately inside the en-
trance, but outside the ward corridor is a bath-roomjfor bathing
patients on admission. The staircase in each case is built
round a lift which is encased with a solid wall on three sides,
a most objectionable arrangement. Wherever lifts must be
placed inside a building of this kind they ^should be enclosed
in skeleton framework only; anything of a more substantial
nature only forms an enclosed shaft, which harbours dust
and serves no purpose whatever, except to facilitate the
passage of air from one ward to another. Close to the
entrance to the ward is a ward kitchen, an isolation ward
for four beds, and a linen room. The large wards
are 101 ft. long, by 25 ft. wide. They contain 32 beds
each, arranged in pairs between the windows. The clear
height of the wards is 12 ft. 3 in., the floor space is about
75 ft., and the cubic space nearly 900 ft. per bed. Each ward
is warmed by means of two stoves with vertical flues,
running upwards through the middle of the wards.
These stoves are supplied with fresh air from the outside,
and the warmed air is admitted into the ward by gratings at
the side of the stoves. To supplement the stoves hot water-
pipes are provided along the two Bide walls. In addition to
the windows, there are in each ward six ventilation beams for
extraction of vitiated air at the ceiling level, and ventilators
under and over the windows. The upper floors are in every
respect similar to the ground floor, except that the isolation
warda are larger by the size of the bath-rooms, which occur
only on the ground floor. The water-closets and sinks are
placed in a tower projected anglewise from one corner of the
ward, the corresponding tower on the other angle containing
bath-rooms and lavatories. Between these towers are covered
balconies entered from the ward. The laundry occupies a
small plot of ground projecting into the workhouse grounds.
It is a one-storey building with a basement in which are
placed the engine-room and workshop. The laundry com-
prises separate washhouse for patients' clothes, for officers'
clothes, and for foul linen ; a general ironing room, a dry-
ing room, and a sorting and delivery room. On the east
side of the site is a post-mortem room and mortuary. The
exterior of the building is finished in a plain and substan-
tial but not ineffective manner, with ordinary brickwork with
a small admixture of stone.

				

## Figures and Tables

**Figure f1:**